# Changes in and Effects of Foreign Language Classroom Anxiety and Listening Anxiety on Chinese Undergraduate Students’ English Proficiency in the COVID-19 Context

**DOI:** 10.3389/fpsyg.2021.670824

**Published:** 2021-05-31

**Authors:** Meihua Liu, Renqing Yuan

**Affiliations:** ^1^Department of Foreign Languages and Literatures, Tsinghua University, Beijing, China; ^2^Beijing University of Posts and Telecommunications, Beijing, China

**Keywords:** foreign language classroom anxiety, foreign language listening anxiety, longitudinal survey study, COVID-19, English proficiency

## Abstract

The present longitudinal survey study explored changes in and effects of foreign language classroom anxiety (FLCA) and listening anxiety (FLLA) on Chinese undergraduate students’ English proficiency over a semester in the COVID-19 context. A set of 182 matching questionnaires was collected from first-year undergraduate English as a foreign language learners at two time points of a 16-week semester. Analyses of the data revealed the following major findings: (1) the participants experienced high levels of FLCA and FLLA both at the beginning and end of the semester, neither of which changed significantly during the semester, (2) FLCA and FLLA were highly positively related to each other, (3) FLCA and FLLA significantly predicted students’ self-rated proficiency in listening and speaking English, and (4) confidence in using English, efforts and motivation to learn English and interaction with instructors and peers mediated FLCA and FLLA to exert effects on students’ self-perceived proficiency in listening and speaking English. These findings indicate that the learning environment is critical in influencing the levels of and changes in FLCA and listening anxiety and that these two types of foreign language anxiety are serious issues in the pandemic foreign language learning context.

## Introduction

Foreign language anxiety is a negative side of emotion specifically related to second/foreign language learning (MacIntyre and Gardner, 1994) and has received considerable attention from researchers in the field (MacIntyre, 2017). As a specific type of foreign language anxiety, foreign language classroom anxiety (FLCA) has been widely researched and proved to be predominantly negatively associated with second/foreign language learning processes or outcomes (e.g., Horwitz et al., 1986; Young, 1986; Aida, 1994; Gregersen and Horwitz, 2002; Botes et al., 2020; Gregersen, 2020; Liu, 2006; Liu and Jackson, 2008). These studies have also revealed that speaking is the most anxiety-provoking activity in second/foreign language learning. By contrast, also as specific types of foreign language anxiety, language skill-related anxieties like listening anxiety, reading anxiety and writing anxiety have been much less investigated in spite of their existence in second/foreign language learning (Phillips, 1992; Cheng et al., 1999; Elkhafaifi, 2005; Jee, 2016). Of the four language skills, listening is viewed as the most frequently used language skill (Scarcella and Oxford, 1992), but “is probably the least explicit of the four language skills, thus, making it the more difficult skill to learn” (Vandergrift, 2004, p. 1). Consequently, foreign language listening is rather challenging and anxiety-provoking to second/foreign language learners (Elkhafaifi, 2005; Liu, 2016). Nevertheless, though foreign language listening anxiety (FLLA) has received more researchers’ attention and been found to be negatively associated with learners’ listening performance (e.g., Elkhafaifi, 2005; Golchi, 2012; Zhang, 2013; Liu, 2016), it still remains under-researched.

As reviewed below, Chinese learners of English at different educational levels often experience anxiety to varying degrees, which is largely because they have limited use of English in daily life (Liu, 2006, 2016; Lu and Liu, 2015; Liu and Xiangming, 2019). They may experience greater anxiety in online learning classrooms when they have even less use of English due to limited interaction and low motivation (Chen, 2010; O’Doherty et al., 2018). Since the outbreak of the pandemic COVID-19 in late 2019, schools in many countries at all levels, including China, are closed and shift to online teaching and learning. Facing this unexpectedly new context, students may encounter numerous challenges and experience various feelings they have seldom had before (Bryson and Andres, 2020). It is thus necessary to examine anxiety in Chinese English as a foreign language learners in this COVID-19 context.

Meanwhile, although the current literature shows that foreign language anxiety, including FLCA and FLLA, is dynamic and multifaceted, studies on changes in different types of anxiety are still inadequate (Liu and Xiangming, 2019; Gregersen, 2020). Even fewer studies are available on the interaction of FLCA and FLLA though they have been shown to be independent constructs of foreign language anxiety (Horwitz et al., 1986; Elkhafaifi, 2005; Bekleyen, 2009).

For these reasons, the present longitudinal survey study aims to examine changes in FLCA and FLLA and their effects on Chinese undergraduates’ speaking and listening proficiency in English in online English language class over a 16-week semester in the COVID-19 context.

## Literature Review

### Foreign Language Classroom Anxiety

Initially treated as a psychological construct, anxiety refers to “the subjective feeling of tension, apprehension, nervousness, and worry associated with an arousal of the autonomic nervous system” and generally has three types: Trait anxiety, state anxiety and situation-specific anxiety (Spielberger, 1983, p. 1). As a type of situation-specific anxiety, foreign language anxiety concerns “the feeling of tension and apprehension specifically associated with second language contexts, including speaking, listening, and learning” (MacIntyre and Gardner, 1994, p. 284). Due to the lack of uniform measures, early studies (e.g., Chastain, 1975; Kleinmann, 1977) revealed inconsistent findings about the role of anxiety in second/foreign language learning. To solve this problem, Horwitz et al. (1986) proposed the FLCA theory, according to which FLCA is “a distinct complex of self-perceptions, beliefs, feelings and behaviors related to classroom learning arising from the uniqueness of the language learning process” (p. 128). To measure this situation-specific type of foreign language anxiety, Horwitz et al. (1986) developed a 33-item Foreign Language Classroom Anxiety Scale covering three dimensions: Communication apprehension, fear of negative evaluation, and test anxiety. Soon, the scale has been widely used by researchers worldwide to explore the relationship between FLCA and second/foreign language learning outcomes measured by language achievement/performance tests, course grades, and/or self-ratings (e.g., Young, 1986; Aida, 1994; Cheng et al., 1999; Matsuda and Gobel, 2004; Liu and Jackson, 2008; Liu and Huang, 2011; Liu and Cheng, 2014; Piniel and Csizér, 2015; Boudreau et al., 2018; Liu, 2018; Liu and Xiangming, 2019; Xiangming et al., 2020; Shirvan and Taherian, 2021). In addition to the consistently negative correlation between FLCA and second/foreign language learning outcomes, these studies have discovered that students are most anxious about speaking the target language in classrooms. Furthermore, these studies, together with those using other measures such as interviews and diaries (e.g., Bailey, 1983; Liu, 2006; Bekleyen, 2009; Jee, 2016), have shown that FLCA levels differ not only in different second/foreign language contexts but also in the same second/foreign language contexts over time, because it continuously interacts with various variables such as familiarity with peers, use of the target language, motivation, enjoyment and self-confidence. For example, using the idiodynamic method, Gregersen et al. (2014) studied three high- and three low-anxiety learners of Spanish as a foreign language via videotaped presentations, self-ratings of anxiety levels and interviews. The results showed many fluctuations in the participants’ anxiety levels during their presentations. Kruk (2018) investigated the changes in the levels of FLCA of 52 Polish senior high school leaners of English over one school semester. Analyses of the collected questionnaires, interviews and lesson plans revealed that FLCA changed not only over the whole semester but also during a single class and from one language lesson to another. Liu and Cheng (2014) examined the relationship between foreign language anxiety and motivation of first-year university students. They found that anxiety levels were significantly lower when students had a higher degree of motivation, which was especially evident among advanced-level students.

Clearly, FLCA, with a focus on speaking, has been much researched: It is existent in many second/foreign language learners and largely negatively affects their learning of the language, and it is changeable during the learning process because of various reasons and interacts with other variables such as motivation and strategy use to affect learning outcomes. Seemingly, learners may also experience other types of anxiety during the learning process like listening anxiety, reading anxiety and writing anxiety, which, nevertheless, have been much less investigated (Phillips, 1992; Cheng et al., 1999; Elkhafaifi, 2005; Jee, 2016). Even fewer studies can be found on the interaction of FLCA and other types of anxiety.

### Foreign Language Listening Anxiety

As more research has been conducted within the FLCA theory, researchers are more aware of the need to examine other types of foreign language anxiety, especially language skills-related anxiety such as listening anxiety, speaking anxiety, writing anxiety and reading anxiety. Consequently, efforts have been made to investigate these types of anxieties (e.g., Sellers, 2000; Gregersen and Horwitz, 2002; Cheng, 2004; Elkhafaifi, 2005; Horwitz et al., 2010; Pae, 2013; Lu and Liu, 2015), which have been shown to be distinct and independent from FLCA and consistently negatively related to the specific language skills and second/foreign language learning outcomes.

Though speaking is widely acknowledged to be the most anxiety-provoking activity in second/foreign language learning (Horwitz et al., 1986; Liu and Jackson, 2008), listening is also a very stressful task for second/foreign language learners (Arnold, 2000). Also as a type of situation-specific anxiety, FLLA refers to the tendency that foreign language listeners become anxious in listening-related tasks (Zhang, 2013; Liu, 2016). Studies have evidenced that FLLA is distinguishable from general foreign language anxiety but positively correlated with it (Kim, 2002; Elkhafaifi, 2005; Bekleyen, 2009; Wang, 2010; Golchi, 2012; Serraj and Noordin, 2013). Studies have also revealed a significantly negative association of FLLA with foreign language listening performance (Elkhafaifi, 2005; Kim, 2002; Mills et al., 2006; Golchi, 2012; Serraj and Noordin, 2013; Zhang, 2013; Liu, 2016; Wang and Cha, 2019). For example, Elkhafaifi (2005) examined the effects of general foreign language learning anxiety and FLLA of 233 postsecondary students of Arabic as a foreign language on their final grades and listening comprehension scores. The results showed that both types of anxiety negatively affected students’ listening comprehension and final grades.

Meanwhile, the current literature shows that FFLA interacts with various variables such as language proficiency, strategy use, self-efficacy and motivation to collaboratively affect listening learning outcomes, as does FLCA (e.g., Kim, 2002; Mills et al., 2006; Golchi, 2012; Liu, 2016; Xu and Huang, 2018; Wang and Cha, 2019). For example, learners with high self-efficacy in their listening capabilities become less anxious while listening to a foreign language (Kim, 2002; Mills et al., 2006). Liu (2016) investigated the relationship between FLLA and strategy use and their predicting effects on test performance of Chinese university students at different English proficiency levels. The results showed that FLLA and strategy use were closely correlated but FLLA was not a powerful predictor for listening test performance when strategy use was considered. Wang and Cha (2019) examined the FLLA of 78 English majors of different English proficiency levels and its effect on their listening performance. They found that English proficiency mediated the effect of FLLA on students’ listening performance. Listening anxiety was a negative predictor while self-belief was a positive predictor for less proficient listeners’ listening performance. Yet these two factors had no predicting power for the high-proficient group’s listening performance.

On the one hand, research on FLLA is relatively inadequate in spite of increasing attention to the issue. On the other hand, little research can be found on changes in FLLA over time though FLLA should be dynamic as well, just like FLCA. Thus, longitudinal studies are needed to examine the dynamic nature of FLLA in various language learning contexts. In addition, since speaking and listening are often intertwined in language learning, FLCA and FLLA may mutually affect each other, as implied in the reviewed literature (Bailey, 1983; Kim, 2002). This, yet, remains to be researched.

### Research on Foreign Language Anxiety in Online Learning Environments

In spite of the plethora of studies on anxiety in various second/foreign language learning contexts, few such studies have been done in online learning environments (Hurd, 2007; Hurd and Xiao, 2010). Online learning is the form of education that takes place over the Internet, where students engage with instructors and other students at their convenient time and place (Singh and Thurman, 2019). Development in information and communications technology eliminates the barriers of time, space, and pace, creating greater flexibility in learning and teaching activities (Aparicio et al., 2017). Nevertheless, some researchers are skeptical about online teaching and learning in that students may experience isolation and struggle to stay motivated, to have adequate face-to-face interaction and get timely responses and feedback (Chen, 2010; O’Doherty et al., 2018). All these may hurt their sense of belonging and confidence in learning (Lei and Gupta, 2010; De Paepe et al., 2018; Janse van Rensburg, 2018). All these are especially important for second/foreign learners, which requires repeated exposure to and practice of the target language in various forms to learn it well.

In terms of foreign language anxiety in online learning environments, studies have revealed different findings (Hurd, 2007; Coryell and Clark, 2009; Hurd and Xiao, 2010; Côté and Gaffney, 2018), which might be due to different learning tasks used in the research. For example, Hurd and Xiao (2010) found that Chinese university students of distance learning were slightly anxious when learning English and felt more anxious when using vocabulary and speaking English. Similarly, the twelve interviewees in Coryell and Clark (2009) remained anxious in the online setting because they focused too much on correctness and precision of their language performance. Côté and Gaffney (2018) explored the effect of typed synchronous computer-mediated communication on foreign language anxiety and output quantity of 61 beginner French learners. They found that compared with traditional classrooms, the participants felt significantly less anxious and produced more conversation turns and words in the online learning context.

Meanwhile, Hurd (2007) investigated changes in anxiety in 500 students enrolled in an online lower-intermediate French course over a 4-month period. The results showed that more than half students’ anxiety levels remained unchanged and some learners tended to be more anxious after 4 months of study. The study also discovered that the participants’ anxiety mainly arose from speaking, such as being called to speak or being fearful of not being understood. Other sources of anxiety included lack of instant feedback, difficulty in assessing personal progress in comparison with other students, isolation, lack of opportunities for speaking practice, lack of confidence when working on their own, and lack of task instructions. Kaisar and Chowdhury (2020) explored whether technology-based virtual classroom could free foreign language learners from anxiety. Analyses of the data collected from a self-developed questionnaire and interviews demonstrated that most students felt more comfortable in face-to-face classrooms than in virtual classrooms. The participants reported that virtual study could not give them opportunities for interactive activities, which made the language class boring and unfruitful. In addition, they attributed their anxiety to such factors as fear of being disconnected, fear of being isolated, lack of interaction with teachers and peers, and network problems.

### Context

To respond to CONVID-19 since its outbreak, the Ministry of Education in China launched an emergency policy initiative entitled “Disrupted Classes, Undisrupted Learning,” to close schools but continue the process of teaching and learning by providing flexible online education via major online platforms like Zoom and Tencent to over 200 million students (Wang et al., 2020). All teaching and learning were conducted online for the first semester of the year 2020 and half online and half in classrooms for the second semester of the year. Thus, how students feel about English speaking and listening in online foreign language classrooms and how this affects their proficiency in speaking and listening English in this special period deserve research, which is the focus of the present research. Targeting Chinese first-year undergraduate students, the present research aims to answer the following research questions:

(1) How do students’ foreign language classroom anxiety and listening anxiety change over the semester?
(2) How are students’ foreign language classroom anxiety and listening anxiety correlated with each other?
(3) How do students’ foreign language classroom anxiety and listening anxiety affect their proficiency in speaking English?
(4) How do students’ listening anxiety and foreign language classroom anxiety affect their proficiency in listening English?

## The Present Study

This study was conducted in a public university in Beijing, where an English language course was mandatory for all first- and second-year undergraduate students, aiming to enhance their listening, speaking, reading and writing skills. As newcomers of the university, more than half first-year students took the English Listening and Speaking course. Coupled with the fact that they were new to the online teaching and learning environment due to COVID-19 in the university and thus might experience greater anxiety than their peers in senior years of study, they became the target population of the present research.

### Participants

Altogether 182 (137 male and 45 female) first-year undergraduate students registered in the English Listening and Speaking course participated in the present study. With an average age of 18.15 (*SD* = 0.70) and an age range of 17–21, the respondents majored in various areas such as electric engineering, computer science, artificial intelligence, information technology, internet security and information communication.

### Instruments

The respondents answered a battery of questionnaires: The background information questionnaire, the Foreign Language Classroom Anxiety Scale, the Foreign Language Listening Anxiety Scale, and self-rated English proficiency and learning of English, as detailed below.

#### Background Information Questionnaire

The Background Information Questionnaire sought to collect such information about the respondents as name, gender, discipline, age, and average use of English per day. Meanwhile, the informants were asked to self-rate their learning of English on the scale of 1 to 10: Ease of searching for materials in English, ease of communicating with others in English, ease of looking for help with English learning, interest in learning English, motivation to learn English, efforts to learn English, confidence in using English, interaction with peers, and interaction with the course instructor. As reported in Table 1, the students rated their learning of English at a similar level in all aspects in both phases.

**TABLE 1:** General information about the participants (*N* = 182).

Self-rated learning of English	Phase 1		Phase 2		Paired samples *t*-test results	
	Mean	*SD*	Mean	*SD*	*t*	*p*
Average use of English per day	0.895	0.69	1.02	1.57	–1.232	0.220
Ease of searching for materials in English	5.05	2.26	4.99	2.46	–0.176	0.860
Ease of communicating with others in English	4.30	2.23	4.52	2.38	–1.014	0.312
Ease of looking for help with English learning	5.02	2.31	5.12	2.39	–1.105	0.271
Interest in learning English	5.56	2.29	5.74	2.53	–0.954	0.341
Motivation to learn English	5.50	2.25	5.55	2.45	–0.022	0.982
Efforts to learn English	4.48	2.02	4.35	2.21	0.717	0.474
Confidence in using English	4.30	2.34	4.70	2.38	–1.447	0.150
Interaction with peers	3.96	2.23	4.36	2.54	–1.561	0.120
Interaction with the English teacher	3.51	2.17	3.91	2.32	–0.990	0.324

#### Foreign Language Classroom Anxiety Scale

With a reliability score of 0.947 in phase 1 and 0.949 in phase 2, this 33-item Foreign Language Classroom Anxiety Scale (FLCAS) was adapted from that developed by Horwitz et al. (1986), primarily aiming to measure students’ anxiety levels in English language classrooms, as done in similar studies (e.g., Hurd and Xiao, 2010; Liu and Xiangming, 2019; Xiangming et al., 2020). To better suit the present situation, expressions ‘foreign languages’ were changed to be ‘English.’

#### Foreign Language Listening Anxiety Scale

Achieving a reliability score of 0.896 in phase 1 and 0.905 in phase 2, the 20-item Foreign Language Listening Anxiety Scale (FLLAS) used in the present study was adopted from that used in Zhang (2013), aiming to measure respondents’ English listening anxiety.

All the FLCAS and FLLAS items were placed on a 5-point Likert scale ranging from ‘Strongly Disagree’ to ‘Strongly Agree’ with values 1–5 assigned to each of the descriptors respectively.

### Self-Rated English Proficiency

The respondents were asked to self-rate their proficiency in English listening and speaking on the scale of 1 (lowest) to 10 (highest), as done in Young (1986) and Liu (2018).

### Procedure

After the design was approved by the Research Committee of the department and the university, the data were collected at two time points over a 13-week period in a 16-week semester. The battery of questionnaires and a consent form were distributed online to around eight natural intact classes of first-year undergraduate students enrolled in the English Listening and Speaking course in week 2 (phase 1), which yielded 261 valid questionnaires. The same questionnaires were distributed to the same students again in week 15 (phase 2), which resulted in 193 questionnaires. Then, the two sets of questionnaires were compared and those with no matching in either phase were deleted, leaving 182 sets of complete matching data in both phases for further analyses.

### Data Analyses

All the data were analyzed via SPSS 20. The FLCAS and FLLAS were first subjected to rotated (varimax) principal factor analysis in both phases to determine their underlying components. Correlation analyses (Pearson two-tailed) were run to examine the correlations between them. Paired samples *t*-tests were then conducted on FLCAS and FLLAS scales to examine changes in FLCA and listening anxiety in two phases respectively. Finally, multiple regression analyses were run to investigate the effects of FLCA, FLLA and self-rated learning of English on students’ self-rated proficiency in English listening and speaking in both phases respectively.

## Results

Prior to any statistical analysis, the adapted FLCAS and FLLAS were subjected to rotated (varimax) principal components analysis in both phases, as done in many existing studies (e.g., Liu and Huang, 2011; Zhang, 2013; Liu and Xiangming, 2019; Wang and Cha, 2019) which sometimes reveal different components in different situations.

The analysis of FLCAS yielded seven factors in phase 1 and six factors in phase 2, with all eigenvalues exceeding 1 (see Appendix Table A1). Based on these results and those of the reviewed literature, coupled with a careful examination of each FLCAS item, the present study adopted a 6-factor solution on the FLCAS, which was then applied to the analyses of the FLCAS in both phases (Appendix Table A2). These six factors were: 12-item FLCAS1 reflective of anxiety about speaking English, nine-item FLCAS2 indicative of worry about the English class, four-item FLCAS3 suggestive of worry about classroom performance, three-item FLCAS4 concerned about anxiety about not understanding the teacher, three-item FLCAS5 reflective of worry about tests, and two-item FLCAS6 indicating worry about mistakes. With reference to Zhang (2013), the analysis of FLLAS resulted in four factors in both phases (see Appendix Table A1): nine-item FLLAS1 reflective of anxiety about listening to English, six-item FLLAS2 suggestive of attitudes toward English listening, three-item FLLAS3 concerned with English listening decoding skills, and two-item FLLAS4 related to English culture in learning listening English.

As shown in Appendices Table A2, A3, the FLCAS items were highly related to the factors they belonged to, so were the FLLAS items. These FLCAS and FLLAS scales were then used for further analyses in the present study. When computing the scores, items reflective of little/no anxiety about or confidence in speaking or listening to English were reverse-coded. Consequently, the higher the FLCAS/FLLAS score, the more anxious a respondent was about speaking/listening to English.

### Changes in Foreign Language Classroom Anxiety and Listening Anxiety

As shown in Table 2, the students scored 3.08 to 3.25 in phase 1 and 3.08 to 3.23 in phase 2 on FLCAS scales, higher than the scale midpoint 3, indicating that more than half of the participants were anxious about speaking English (FLCAS1), worried about the English class (FLCAS2) and classroom performance (FLCAS3), anxious about not understanding the teacher (FLCAS4), and worried about tests (FLCAS5) and mistakes (FLCAS6) in both phases. Similarly, they scored 2.89–3.29 in phase 1 and 2.97–3.23 in phase 2 on FLLAS scales, around the scale midpoint 3, meaning that around half of the participants were anxious about listening to English (FLLAS1), held negative attitudes toward English listening and were not satisfied with their English listening proficiency (FLLAS2), were poor in decoding English listening (FLLAS3) and worried about learning English culture in order to learn English listening well (FLLAS4) in both phases.

**TABLE 2:** Means, standard deviations, and paired samples *t*-test results of FLCAS and FLLAS scales in both phases (*N* = 182).

	Phase 1		Phase 2		Paired samples *t*-test results	
					
	Mean	*SD*	Mean	*SD*	*t*	*P*
FLCAS1	3.25	0.72	3.18	0.75	1.248	0.213
FLCAS2	3.08	0.65	3.08	0.74	0.518	0.60
FLCAS3	3.18	0.89	3.11	0.92	1.08	0.281
FLCAS4	3.13	0.898	3.08	0.93	1.521	0.130
FLCAS5	3.22	0.85	3.23	0.88	0.094	0.925
FLCAS6	3.24	0.77	3.23	0.92	0.301	0.764
FLCAS	3.18	0.65	3.14	0.71	1.001	0.318
FLLAS1	2.98	0.76	3.01	0.82	0.227	0.821
FLLAS2	3.14	0.62	3.06	0.59	1.63	0.105
FLLAS3	3.29	0.87	3.23	0.94	1.124	0.262
FLLAS4	2.89	0.67	2.97	0.72	–1.306	0.193
FLLAS	3.06	0.60	3.05	0.65	0.676	0.500
Listening	4.11	2.21	4.11	2.18	0.045	0.964
Speaking	3.95	2.16	4.21	2.22	–0.755	0.451

*FLCAS1, anxiety about speaking English; FLCAS2, worry about the English class; FLCAS3, worry about classroom performance; FLCAS4, anxiety about not understanding the teacher; FLCAS5, worry about tests; FLCAS6, worry about mistakes; FLCAS, Foreign Language Classroom Anxiety Scale; FLLAS1, anxiety about listening to English; FLLAS2, attitudes toward English listening; FLLAS3, English listening decoding skills; FLLAS4, worry about English culture related to listening English; FLLAS, Foreign Language Listening Anxiety Scale; Listening, self-rated listening proficiency; Speaking, self-rated speaking proficiency.*

As seen from Table 2, the students tended to score lower or similar on FLCAS and FLLAS scales in phase 2. Nevertheless, no statistically significant differences occurred in any of the scales, as evidenced by paired samples t test results reported in Table 2.

Meanwhile, Table 2 shows that the informants self-rated their English listening proficiency as 4.11 in both phases and English speaking proficiency as 3.95 in phase 1 and 4.21 in phase 2, quite low on the scale of 1 to 10. And no significant increase or decrease in the students’ self-rated proficiency in English listening or speaking occurred in phase 2.

### Correlations Between FLCAS and FLLAS Scales

As reported in Table 3, FLCAS and FLLAS scales were significantly positively related to one another in both phases (*r* = 0.244 ∼0.819 in phase and 0.248 ∼0.826 in phase 2, *p* ≤ 0.002). Alternatively, the higher the FLCAS score, the higher the FLLAS score. For example, the more anxious a respondent was about speaking English (FLCAS1), the more anxious he/she was about listening to English (FLLAS1) (*r* = 0.695 in phase 1 and 0.715 in phase 2).

**TABLE 3:** Correlations between FLCAS and FLLAS scales in both phases (*N* = 182).

	FLLAS1	FLLAS2	FLLAS3	FLLAS4	FLLAS
FLCAS1	0.695**/0.715**	0.611**/0.599**	0.599**/0.613**	0.314**/0.266**	0.749**/0.738**
FLCAS2	0.669**/0.684**	0.638**/0.723**	0.600**/0.651**	0.244**/0.274**	0.735**/0.763**
FLCAS3	0.691**/0.733**	0.591**/0.581**	0.590**/0.662**	0.297**/0.441**	0.737**/0.773**
FLCAS4	0.703**/0.705**	0.397**/0.442**	0.540**/0.609**	0.386**/0.224**	0.682**/0.683**
FLCAS5	0.588**/0.643**	0.522**/0.538**	0.558**/0.650**	0.277**/0.375**	0.648**/0.700**
FLCAS6	0.450**/0.484**	0.415**/0.424**	0.394**/0.408**	0.209**/0.248**	0.493**/0.511**
FLCAS	0.765**/0.782**	0.657**/0.676**	0.663**/0.705**	0.337**/0.339**	0.819**/0.826**

The first number refers to the coefficient in phase 1 and second refers to the coefficient in phase 2. **p ≤ 0.002; coefficient of determination: small = *r* ≤ 0.1; medium = *r* = 0.3; large = *r* ≥ 0.5 (Cohen, 1988).

### Effects of FLCA and FLLA on Self-Rated Proficiency in Speaking English

In order to explore the effects of FLCA and FLLA on English speaking proficiency, multiple stepwise regression analyses were conducted three times in both phases respectively, with self-rated English speaking proficiency as the dependent variable, FLCAS scales as independent variables the first time, FLCAS and FLLAS scales as independent variables the second time, and FLCAS and FLLAS scales and self-rated learning of English as independent variables the third time. The results are summarized in Tables 4, 5.

**TABLE 4:** Multiple regression coefficients and significance of predictors for speaking English proficiency (phase 1).

		FLCAS	FLCAS4				
Speaking: FLCAS as independent variables	β	–0.806	0.307				
	*t*	–11.43**	4.36**				
	*p*	0.000	0.000				
	VIF	2.09	2.09				
	Cohen’s *f*^2^	0.12	0.045				

		**FLCAS**	**FLCAS4**	**FLLAS**	**FLLAS4**		

Speaking: FLCAS and FLLAS as independent variables	β	–0.557	0.341	–0.388	0.132		
	*t*	–6.11**	4.81**	–4.23**	2.36*		
	*p*	0.000	0.000	0.000	0.019		
	VIF	3.72	2.25	3.768	1.392		
	Cohen’s *f*^2^	0.12	0.045	0.029	0.012		

		**Confidence in using English**	**FLLAS**	**Efforts to learn English**	**FLLAS4**	**FLCAS4**	**FLCAS1**

Speaking: All variables as independent variables	β	0.452	–0.312	0.137	0.122	0.164	–0.152
	*t*	7.23**	–4.24**	2.83**	2.59**	2.88**	–2.20*
	*p*	0.000	0.000	0.005	0.01	0.004	0.029
	VIF	2.43	3.369	1.459	1.373	2.015	2.95
	Cohen’s *f*^2^	0.27	0.026	0.013	0.014	0.009	0.008

**p ≤ 0.01; *p ≤ 0.05. Effect size of Cohen’s *f*^2^: small = *f*^2^ ≤ 0.02; medium = *f*^2^ = 0.15; large = *f*^2^ ≥ 0.35 (Cohen, 1988).

**TABLE 5:** Multiple regression coefficients and significance of predictors for speaking English proficiency (phase 2).

		FLCAS		
Speaking: FLCAS as independent variables	β	–0.503		
	*t*	–7.80**		
	*p*	0.000		
	VIF	1.000		
	Cohen’s *f*^2^	0.064		

		**FLCAS**	**FLLAS2**	**FLLAS**

Speaking: FLCAS and FLLAS as independent variables	β	–0.465	–0.391	0.274
	*t*	–4.20**	–3.95**	2.12*
	*p*	0.000	0.000	0.035
	VIF	3.172	2.536	0.432
	Cohen’s *f*^2^	0.064	0.043	0.017

		**Interaction with**	**FLCAS**	**FLLAS2**

		**instructor**		
Speaking: All variables as independent variables	β	0.361	–0.248	–0.184
	*t*	5.68**	–3.13**	–2.29*
	*p*	0.000	0.002	0.023
	VIF	1.203	1.881	1.929
	Cohen’s *f*^2^	0.074	0.114	0.018

**p ≤ 0.01; *p ≤ 0.05. Effect size of Cohen’s *f*^2^: small = *f*^2^ ≤ 0.02; medium = *f*^2^ = 0.15; large = *f*^2^ ≥ 0.35 (Cohen, 1988).

As shown in Table 4 (phase 1), when FLCAS scales were used as independent variables, FLCAS (overall FLCA) and FLCAS4 (anxiety about not understanding the teacher) were powerful predictors for self-rated English speaking proficiency, with FLCAS being a negative (β = –0.806, *t* = –11.43, *p* = 0.000) and FLCAS4 a positive (β = 0.307, *t* = 4.36, *p* = 0.000) predictor. When FLCAS and FLLAS scales were used as independent variables, FLCAS, FLCAS4, FLLAS (overall FLLA) and FLLAS4 (worry about English culture related to listening English) were powerful predictors for self-rated English speaking proficiency. FLCAS (β = –0.557, *t* = –6.11, *p* = 0.000) and FLLAS (β = –0.388, *t* = –4.23, *p* = 0.000) were negative predictors while FLCAS4 (β = 0.341, *t* = 4.81, *p* = 0.000) and FLLAS4 (β = 0.132, *t* = 2.36, *p* = 0.019) were positive predictors. When FLCAS and FLLAS scales and self-rated learning of English were used as independent variables, confidence in using English (β = 0.452, *t* = 7.23, *p* = 0.000), FLLAS (β = –0.312, *t* = –4.24, *p* = 0.000), efforts to learn English (β = 0.137, *t* = 2.83, *p* = 0.005), FLLAS4 (β = 0.122, *t* = 2.59, *p* = 0.01), FLCAS4 (β = 0.164, *t* = 2.88, *p* = 0.004), and FLCAS1 (anxiety about speaking English) (β = –0.152, *t* = –2.20, *p* = 0.029) proved to be powerful predictors for self-rated English speaking proficiency.

As reported in Table 5, when FLCAS scales were used as independent variables, FLCAS (β = –0.503, *t* = –7.80, *p* = 0.000) was a powerful negative predictor for self-rated English speaking proficiency. When FLCAS and FLLAS scales were used as independent variables, FLCAS (β = –0.465, *t* = –4.20, *p* = 0.000), FLLAS2 (attitudes toward English listening) (β = –0.391, *t* = –3.95, *p* = 0.000), and FLLAS (β = 0.274, *t* = 2.12, *p* = 0.035) were powerful predictors for self-rated English speaking proficiency. FLCAS and FLLAS2 were negative predictors while FLLAS was a positive one. When FLCAS and FLLAS scales and self-rated learning of English were used as independent variables, interaction with the course instructor (β = 0.361, *t* = 5.68, *p* = 0.000), FLCAS (β = –0.248, *t* = –3.13, *p* = 0.002), and FLLAS2 (β = –0.184, *t* = –2.29, *p* = 0.023) proved to be powerful predictors for self-rated English speaking proficiency.

### Effects of FLCA and FLLA on Self-Rated Proficiency in Listening English

In order to explore the effects of FLCA and FLLA on English listening proficiency, multiple stepwise regression analyses were conducted three times in both phases respectively, with self-rated English listening proficiency as the dependent variable, FLLAS scales as independent variables the first time, FLLAS and FLCAS scales as independent variables the second time, and FLLAS and FLCAS scales and self-rated learning of English as independent variables the third time. The results are summarized in Tables 6, 7.

**TABLE 6:** Multiple regression coefficients and significance of predictors for listening English proficiency (phase 1).

		FLLAS	FLLAS4	FLLAS1	
Listening: FLLAS as independent variables	β	–1.103	0.236	0.508	
	*t*	–7.02**	3.93**	3.38**	
	*p*	0.000	0.000	0.001	
	VIF	9.33	1.36	8.54	
	Cohen’s *f*^2^	0.067	0.032	0.30	

		**FLLAS**	**FLLAS4**	**FLLAS1**	**FLCAS3**

Listening: FLLAS and FLCAS as independent variables	β	–0.922	0.214	0.500	–0.220
	*t*	–5.53**	3.59**	3.37**	–2.91**
	*p*	0.000	0.000	0.001	0.004
	VIF	10.83	1.39	8.55	2.22
	Cohen’s *f*^2^	0.067	0.032	0.030	0.022

		**Confidence in using English**	**FLLAS3**	**FLLAS2**	

Listening: All variables as independent variables	β	0.464	–0.164	–0.145	
	*t*	7.78**	–2.92**	–2.38*	
	*p*	0.000	0.004	0.018	
	VIF	1.63	1.46	1.70	
	Cohen’s *f*^2^	0.15	0.033	0.012	

**p ≤ 0.01; *p ≤ 0.05. Effect size of Cohen’s *f*^2^: small = *f*^2^ ≤ 0.02; medium = *f*^2^ = 0.15; large = *f*^2^ ≥ 0.35 (Cohen, 1988).

**TABLE 7:** Multiple regression coefficients and significance of predictors for listening English proficiency (phase 2).

		FLLAS2	FLLAS3		
Listening: FLLAS as independent variables	β	–0.483	–0.147		
	*t*	–6.61**	–2.004*		
	*p*	0.000	0.047		
	VIF	1.437	1.437		
	Cohen’s *f*^2^	0.101	0.015		

		**FLLAS2**	**FLCAS3**		

Listening: FLLAS and FLCAS as independent variables	β	–0.441	–0.212		
	*t*	–5.95**	–2.85**		
	*p*	0.000	0.005		
	VIF	1.510	1.510		
	Cohen’s *f*^2^	0.101	0.030		

		**FLLAS2**	**Motivation to learn English**	**Interaction with peers**	**FLCAS3**

Listening: All variables as independent variables	β	–0.259	0.275	0.190	–0.192
	*t*	–3.46**	4.36**	3.12**	–2.81**
	*p*	0.001	0.000	0.002	0.006
	VIF	1.823	1.288	1.199	1.515
	Cohen’s *f*^2^	0.101	0.078	0.033	0.024

**p ≤ 0.01; *p ≤ 0.05. Effect size of Cohen’s *f*^2^: small = *f*^2^ ≤ 0.02; medium = *f*^2^ = 0.15; large = *f*^2^ ≥ 0.35 (Cohen, 1988).

As shown in Table 6, when FLLAS scales were used as independent variables, FLLAS (β = –1.103, *t* = –7.02, *p* = 0.000), FLLAS4 (β = 0.236, *t* = 3.93, *p* = 0.000), and FLLAS1 (anxiety about listening to English) were (β = 0.508, *t* = 3.38, *p* = 0.001) powerful predictors for self-rated English listening proficiency, with FLLAS being a negative and the latter two positive predictors. When FLLAS and FLCAS scales were used as independent variables, FLLAS, FLLAS4, FLLAS1, and FLCAS3 (worry about classroom performance) were powerful predictors for self-rated English listening proficiency. FLLAS (β = –0.922, *t* = –5.53, *p* = 0.000) and FLCAS3 (β = –0.220, *t* = –2.91, *p* = 0.004) were negative predictors while FLLAS4 (β = 0.214, *t* = 3.59, *p* = 0.000) and FLLAS1 (β = 0.500, *t* = 3.37, *p* = 0.001) were positive predictors. When FLLAS and FLCAS scales and self-rated learning of English were used as independent variables, confidence in using English (β = 0.464, *t* = 7.78, *p* = 0.000), FLLAS3 (English listening decoding skills) (β = –0.164, *t* = –2.92, *p* = 0.004), and FLLAS2 (β = –0.145, *t* = –2.38, *p* = 0.018) proved to be powerful predictors for self-rated English listening proficiency.

As reported in Table 7, when FLLAS scales were used as independent variables, FLLAS2 (β = –0.483, *t* = –6.61, *p* = 0.000) and FLLAS3 (β = –0.137, *t* = –2.004, *p* = 0.047) were powerful negative predictors for self-rated English listening proficiency. When FLLAS and FLCAS scales were used as independent variables, FLLAS2 (β = –0.441, *t* = –5.95, *p* = 0.000) and FLCAS3 (β = –0.212, *t* = –2.85, *p* = 0.005) were powerful negative predictors for self-rated English listening proficiency. When FLLAS and FLCAS scales and self-rated learning of English were used as independent variables, FLLAS2 (β = –0.259, *t* = –3.46, *p* = 0.001), motivation to learn English (β = 0.275, *t* = 4.36, *p* = 0.000), interaction with peers (β = 0.190, *t* = 3.12, *p* = 0.002), and FLCAS3 (β = –0.192, *t* = –2.81, *p* = 0.006) were powerful predictors for self-rated English listening proficiency. FLLAS2 and FLCAS3 were negative while motivation to learn English and interaction with peers were positive predictors.

## Discussion

The present study revealed that both the FLCAS and the FLLAS had underlying components, were highly reliable and significantly positively correlated with each other.

### Changes in Foreign Language Classroom Anxiety and Listening Anxiety

Statistical analyses showed that the students experienced a quite high level of FLCA, higher than did their peers in traditional classrooms in Liu’s studies (Liu, 2006, 2016; Liu and Jackson, 2008; Liu and Xiangming, 2019). Their anxiety was also greater than that experienced by distance learners in Hurd and Xiao (2010), which might be because the participants in the present study were new to the university and the learning environment while those in the latter were not. In addition, the participants in the present study suffered from high FLLA, also higher than did their peers in traditional classrooms in Liu (2016). These findings might be because the participants, as newcomers to the university, were not accustomed to the new environment at the beginning of the semester and still did not adapt themselves to the pandemic online learning context during the semester, where they could not turn to their peers and teachers as easily as in traditional classrooms, as reported in Hurd (2007) and Kaisar and Chowdhury (2020). However, as discussed in Gong et al. (2020a,b, 2021) and Scull et al. (2020), interaction between students and instructors as well as with others is crucial in successfully learning a second/foreign language. This is especially important for online teaching in facilitating and supporting the learning process (Bryson and Andres, 2020). As reported in Coman et al. (2020), lack of interaction was a big problem faced by Romanian university students when teaching became online due to COVID-19. In order to help students learn online better due to the COVID-19 pandemic, teachers in Scull et al. (2020) made efforts to ensure that materials were accessible and responsive to students’ needs, strengthen students’ participation through building relationships and connecting with them, and engage students socially and intellectually. Naturally, these efforts led to good results.

Due to the lack of interaction, the participants might not feel confident and comfortable when learning and using English, as discussed in Chen (2010) and O’Doherty et al. (2018). Another possible reason was that because of the precautions against COVID-19, students could not access libraries as much as they could, nor could they communicate with peers or teachers as much as possible or as easily as they did in normal school learning situations, as reported by the participants in the present study. They even could not easily search for resources on Internet due to various reasons such as cost, instability of the Internet, and so on. All these might have (greatly) hindered them from knowing one another and building a sense of belonging and community. Thus, they were not familiar with their peers, not easy to communicate with others in English or look for help with their English learning, not confident in using English, not interested in learning or motivated to learn English, as discussed in the current literature (Lei and Gupta, 2010; De Paepe et al., 2018; Janse van Rensburg, 2018). They did not make much effort to learn English either, had not much interaction with their peers and instructors, and self-rated their proficiency in listening and speaking English rather low, as reported in Tables 1, 2, both at the beginning and end of the semester. As a result, in both phases, they were apprehensive of speaking and listening to English in class, worried about the English class and classroom performance, were afraid of mistakes and English tests, were not satisfied with their English listening proficiency and tried to translate word by word when listening to English, and were upset by the culture they had to learn to understand spoken English.

All these reasons also largely explained why the participants’ levels of FLCA and listening anxiety generally remained unchanged over the 16-week semester, similar to Hurd’s (2007) study of online learners of French but different from those of learners in traditional classrooms (Gregersen et al., 2014; Kruk, 2018; Liu and Xiangming, 2019; Xiangming et al., 2020). Although this result might partially be due to the fact that relatively a small number of students answered the questionnaires, it indicated that the learning environment was crucial to the levels of and changes in FLCA and FLLA, also evidenced in Gong et al. (2020a,b, 2021) studies in the study-abroad context and those in online or traditional classrooms previously reviewed. Namely, a learning environment facilitates learning if it provides adequate resources and opportunities for students to learn and practice the target language and develop themselves (e.g., building self-confidence, and developing communication skills, etc.) in various ways. However, if learners cannot have more access to and practice of the target language, have little chance of getting familiarized with their peers and course instructors, and have difficulty in getting help for their learning of the target language, they are likely to experience the same level of FLCA and listening anxiety during a long period in the same context.

### Effects of FLCA and FLLA on Self-Rated Proficiency in Speaking and Listening English

In both phases, whether working alone or working with FLLAS scales, FLCAS (overall foreign language anxiety) remained a powerful negative predictor for self-rated speaking English proficiency. FLCAS4 (worry about not understanding the teacher) was a powerful positive predictor in phase 1. When different variables worked together, FLLAS (overall FLLA) remained a powerful negative predictor in all cases in both phases, FLLAS4 (worry about English culture related to listening English) a negative predictor in phase 1 and FLLAS2 (attitudes toward English listening) a negative predictor in phase 2. These findings clearly indicated that FLCA (overall FLCA and anxiety about speaking English) and FLLA greatly affected students’ self-perceived proficiency in speaking English, as found in Liu (2018) and Liu and Xiangming (2019). In particular, incomprehensible input (i.e., not understanding the teacher or the culture related to speaking English) and attitudes toward English listening had special impacts on students’ self-perceived proficiency in speaking English, as discussed in Bailey (1983), Horwitz et al. (1986), and Liu (2006). In addition, confidence in using English, efforts to learn English and interaction with the course instructor mediated FLCAS and FLLAS scales to exert effects on students’ self-perceived proficiency in speaking English, as reported by learners in Bailey (1983), Liu (2006), Bekleyen (2009), and Jee (2016).

Whether working alone or working with other variables, FLLAS (overall FLLA) and/or its subscales significantly predicted students’ self-rated listening English proficiency in all cases in both phases. FLCAS3 (worry about classroom performance) powerfully negatively predicted the latter in both phases. These findings suggest that FLLA (FLLA, negative attitudes toward English learning and poor English listening skills) and worry about classroom performance (FLCAS3) are powerful predictors for students’ self-rated listening English proficiency, as found in Elkhafaifi (2005) and Liu (2016). When different variables worked together, FLLAS1 (worry about English listening) became a positive predictor, indicating that anxiety might facilitate second/foreign language learning to a certain degree, as found in Bailey (1983) and Liu (2006). Moreover, confidence in using English, motivation to learn English and interaction with peers mediated FLCAS and FLLAS scales to exert effects on students’ self-perceived proficiency in listening English, as discussed in Elkhafaifi (2005) and Botes et al. (2020).

Clearly, FLCA and FLLA affected students’ self-perceived proficiency in listening and speaking English respectively. Moreover, FLLA seemed to have a greater effect on self-rated proficiency in speaking English than did FLCA on self-rated proficiency in listening English.

## Conclusion and Implications

The present study investigated changes in and effects of FLCA and listening anxiety on Chinese undergraduate students’ English proficiency over a semester in the COVID-19 context. Analyses of a set of 182 matching questionnaires revealed the following major findings: (1) the respondents reported a similarly high level of FLCA and FLLA both at the beginning and end of the semester, (2) FLCAS and FLLAS scales were highly positively related to one another, (3) FLCA and FLLA significantly predicted the respondents’ self-rated proficiency in listening and speaking English. FLLA seemed to have a greater effect on self-rated proficiency in speaking English than did FLCA on self-rated proficiency in listening English, and (4) confidence in using English, efforts and motivation to learn English and interaction with the English instructor and peers mediated FLCAS and FLLAS scales to affect the participants’ self-rated proficiency in listening and speaking English.

Although the relatively small number of respondents might weaken the generalizability of the findings in the present study, these findings clearly show that the learning environment is critical in influencing the levels of and changes in FLCA and FLLA and that FLCA and FLLA are serious issues in the COVID-19 foreign language learning context. As reported in many current studies (Bailey, 1983; Horwitz et al., 1986; Elkhafaifi, 2005; Liu, 2006, 2018; Liu and Xiangming, 2019), as students have more access to and practice of the target language, become more familiar with their peers, the course instructor and the classroom and school learning environment, they often become more confident and less anxious when using the target language in class. However, due to the fast and widespread of COVID-19, the participants in the present study had limited access to and practice of speaking and listening to English, were distant from their peers and course instructors, and could not resort to many resources from the Internet and school libraries, during the whole semester. Thus, they suffered from quite high levels of FLCA and FLLA both at the beginning and end of the semester, which did not show any significant change. Consequently, how to help reduce students’ FLCA and FLLA levels in the pandemic language learning context is a significant issue. On the part of language instructors, it is useful for them to give a clear description of the course, including requirements, expectations, content, tasks, assessment in relation to the COVID-19 context at the beginning of a semester (Kruk, 2018). This could help relieve students’ worry about the course and the unpredictable new context to a certain degree. During the semester, it is important for instructors to encourage students to speak English to one another and listen to English as much as possible both in and outside the online/traditional classroom. Course instructors had better try all means to build a friendly and supportive classroom atmosphere to help students feel at ease when using English in class, as discussed in the current literature (e.g., Horwitz et al., 1986; Phillips, 1992; Golchi, 2012; Liu, 2018; Liu and Xiangming, 2019). For example, in the listening English class, teachers can first understand students’ listening proficiency level, provide them with comprehensible input to let them experience small success and to boost their confidence, and then offer materials with increasing difficulties (Elkhafaifi, 2005). In addition, as suggested by Oxford (2008), listening instructions can focus on specific listening strategies (e.g., listening to key words, paying attention to word stress) to improve students’ listening skills and on specific positive feedback to increase students’ positive learning experiences and to cultivate their positive attitudes toward foreign language listening class.

Meanwhile, as found in MacIntyre et al. (2020) large-scale survey study, language teachers are also new to and under great stress in this unexpected CONVID-19 pandemic. Thus, it is better for them to spend more time planning and preparing their lessons and reflect their teaching practices and adjust them timely to cater to the new teaching and learning experience imposed by COVID-19, as did Bryson and Andres (2020). To facilitate teaching and help students, it is necessary for teachers to provide both synchronous and asynchronous learning opportunities (Bryson and Andres, 2020; Scull et al., 2020). It is also helpful for them to provide emotional, technical and instructional support for students, actively interact with students and seek support for teaching and learning (Bryson and Andres, 2020; MacIntyre et al., 2020). All these can be easily realized by building relationships and connections via Wechat, email, and blogs as well as classroom interactions.

In addition to actively interacting with their peers and course instructors and engaging themselves in all classroom activities, students themselves should make use of all resources they can find to increase their exposure to and use of English, such as BBC and VOA news, Ted talks and TV episodes (Zhang, 2013; Liu and Xiangming, 2019; Wang and Cha, 2019). Autonomous learning and active responses to the unexpected pandemic are always beneficial. This is best demonstrated by adult New Zealanders learning Chinese as a second language in China in Gong et al. (2020a, 2021), who made strategic efforts to seek opportunities and resources for practicing spoken Chinese outside the classroom. All these efforts can not only help reduce their FLCA and FLLA levels but also improve their proficiency in overall English as well as speaking and listening English. Then, a beneficial circle may form: Lower FLCA and FLLA lead to higher proficiency in speaking and listening English, and vice versa.

The present study enriched the current literature on foreign language anxiety by examining changes in and effects of FLCA and FLLA at different time points in the COVID-19 context. Even so, it can be bettered in several ways. First, the participants in the present study were homogeneous and the sample size was relatively small, which might make the findings less generalizable. Future studies can recruit more participants from diverse populations and thus present a fuller profile of anxiety in pandemic online learning contexts. Second, a deeper understanding of foreign language anxiety in online and traditional learning classrooms can be achieved if future studies can simultaneously cover both situations with triangulated data. The results may lend further support to the finding in Côté and Gaffney (2018) that online learners felt significantly less anxious than those in traditional classrooms. The results may also help identify sources for anxiety in pandemic online learning contexts.

## Data Availability Statement

The raw data supporting the conclusions of this article will be made available by the authors, without undue reservation.

## Ethics Statement

The studies involving human participants were reviewed and approved by Research Committee of the Department of Foreign Languages and Literatures, Tsinghua University. The patients/participants provided their written informed consent to participate in this study.

## Author Contributions

ML contributed to conception and design of the study, performed the statistical analysis, and wrote sections of the manuscript. RY organized the database and wrote sections of the manuscript. Both authors contributed to the manuscript revision, read, and approved the submitted version.

## Conflict of Interest

The authors declare that the research was conducted in the absence of any commercial or financial relationships that could be construed as a potential conflict of interest.

## Appendix

**TABLE A1:** Eigenvalues and explained variances of FLCAS and FLLAS factors.

	Phase 1		Phase 2	
	Eigenvalue	% of total variance	Eigenvalue	% of total variance
FLCAS1	4.199	12.52%	5.853	17.74%
FLCAS2	4.075	12.45%;	4.365	13.23%
FLCAS3	3.319	11.48%	4.284	12.98%
FLCAS4	2.732	9.91%	2.858	8.66%
FLCAS5	2.632	5.20%	1.778	5.39%
FLCAS6	2.007	4.99%	1.667	5.05%
FLCAS7	1.452	3.98%		
FLLAS1			5.597	27.98%
FLLAS2			2.642	13.21%
FLLAS3			1.964	9.82%
FLLAS4			1.753	8.76%

**TABLE A2:** Loadings of principal components of FLCAS (phases 1/2) [Kaiser–Meyer–Olkin (KMO) = 0.942 in phase 1 and 0.921 in phase 2 (*p* ≤ 0.001)].

	Factor 1	Factor 2	Factor 3	Factor 4	Factor 5	Factor 6
(1) I never feel quite sure of myself when I am speaking English in my class.	**0.376/0.254**	0.215/0.574	0.524/–0.359	0.126/0.376	0.200/0.029	0.175/–0.103
(2) I don’t worry about making mistakes in the English class.	–0.253/–0.134	–0.022/–0.431	–0.642/0.568	–0.034/–0.011	0.034/–0.008	**0.066/–0.108**
(3) I tremble when I know that I’m going to be called on in the English class.	**0.724/0.355**	0.085/0.644	0.323/–0.314	–0.076/0.152	0.140/0.053	0.020/–0.037
(4) It frightens me when I don’t understand what the teacher is saying in English.	0.361/0.401	0.580/0.379	0.183/–0.295	**0.093/0.213**	0.055/0.328	–0.035/–0.085
(5) It wouldn’t bother me at all to take more foreign language classes.	–0.060/–0.160	**–0.108/–0.146**	–0.656/0.619	–0.170/–0.223	–0.108/0.009	0.031/0.034
(6) During my English class, I find myself thinking about things that have nothing to do with the course.	0.064/0.212	**0.005/0.071**	–0.030/–0.085	0.067/0.141	0.142/0.185	0.792/0.726
(7) I keep thinking that the other students are better at English than I am.	0.266/0.310	0.328/0.185	**0.480/–0.282**	0.052/0.698	0.518/0.012	0.268/0.217
(8) I am usually at ease during English tests in my class.	–0.247/–0.309	–0.350/–0.041	–0.602/0.634	–0.097/–0.330	–**0.157/**–**0.204**	0.002/0.065
(9) I start to panic when I have to speak without preparation in the English class.	**0.553/0.113**	0.225/0.554	0.231/–0.173	0.258/0.505	0.279/0.217	–0.123/0.000
(10) I worry about the consequences of failing my English class.	0.172/0.271	0.530/0.176	0.234/–0.200	0.100/0.601	**0.368/0.314**	–0.043/–0.040
(11) I don’t understand why some people get so upset over English classes.	–0.198/–0.369	–**0.219/0.027**	–0.588/0.599	–0.087/–0.071	–0.058/0.038	0.034/0.098
(12) In the English class, I can get so nervous I forget things I know.	0.547/0.355	**0.392/0.228**	0.110/–0.120	0.126/0.319	0.101/0.531	0.026/0.263
(13) It embarrasses me to volunteer answers in my English class.	**0.656/0.357**	0.081/0.637	0.122/–0.134	0.186/0.060	0.089/0.270	0.192/0.053
(14) I would not be nervous speaking English with native speakers.	–**0.152/**–**0.158**	–0.106/–0.101	–0.404/0.653	–0.585/0.040	–0.030/–0.402	–0.040/0.119
(15) I get upset when I don’t understand what the teacher is correcting.	0.169/0.227	0.686/0.397	0.114/–0.076	**0.284/0.126**	0.079/0.489	–0.040/0.127
(16) Even if I am well prepared for the English class, I feel anxious about it.	0.604/0.579	**0.225/0.413**	0.289/–0.151	0.101/0.109	0.027/0.247	0.177/0.209
(17) I often feel like not going to my English class.	0.266/0.617	**0.415/0.206**	0.233/–0.176	0.008/–0.187	–0.254/0.144	0.504/0.432
(18) I feel confident when I speak English in class.	–**0.353/**–**0.158**	–0.264/–0.156	–0.515/0.673	–0.245/–0.220	–0.226/–0.058	–0.117/0.034
(19) I am afraid that my English teacher is ready to correct every mistake I make.	0.582/0.310	0.229/0.311	0.129/–0.016	0.140/–0.059	–0.175/0.585	–**0.062/0.087**
(20) I can feel my heart pounding when I’m going to be called on in the English class.	**0.685/0.216**	0.116/0.798	0.162/–0.066	0.095/–0.046	0.299/0.162	–0.101/0.140
(21) The more I study for an English test, the more confused I get.	0.318/0.535	0.470/0.222	0.172/0.024	–0.017/0.356	**0.166/0.020**	0.198/0.311
(22) I don’t feel pressure to prepare very well for the English class.	–0.146/–0.127	–**0.319/0.018**	–0.624/0.618	–0.108/–0.286	–0.363/0.130	–0.006/–0.313
(23) I always feel that the other students speak English better than I do.	0.320/0.305	0.306/0.348	**0.366/**–**0.255**	0.064/0.654	0.557/0.213	0.245/0.104
(24) I feel very self-conscious about speaking English in front of other students.	**0.688/0.291**	0.305/0.529	0.258/–0.201	0.028/0.215	–0.005/0.347	0.139/0.092
(25) The English class moves so quickly I worry about getting left behind.	0.254/0.679	0.537/0.271	**0.364/**–**0.154**	–0.034/0.225	0.229/0.186	0.200/0.077
(26) I feel more tense and nervous in my English class than in my other classes.	0.411/0.771	**0.461/0.315**	0.453/–0.196	–0.106/0.177	–0.178/0.068	0.098/–0.045
(27) I get nervous and confused when I am speaking English in class.	**0.638/0.641**	0.403/0.329	0.262/–0.215	–0.006/0.226	–0.031/0.220	0.257/0.056
(28) When I’m on my way to the English class, I feel very sure and relaxed.	–0.214/–0.274	–**0.113/**–**0.149**	–0.667/0.727	–0.162/–0.014	–0.000/–0.060	–0.247/–0.178
(29) I get nervous when I don’t understand every word the English teacher says.	0.201/0.532	0.712/0.114	0.103/–0.060	**0.130/0.301**	–0.044/0.491	–0.073/–0.338
(30) I feel overwhelmed by the number of rules I have to learn to speak English.	**0.218/0.632**	0.687/0.163	0.098/–0.162	0.104/0.310	0.027/0.262	0.214/0.072
(31) I am afraid that the other students will laugh at me when I speak English.	0.555/0.493	0.348/0.325	**0.204/**–**0.161**	0.062/0.121	0.040/0.414	0.104/0.161
(32) I would probably feel comfortable around native speakers of English.	**0.029/0.182**	–0.098/–0.169	–0.508/0.693	–0.602/0.066	0.125/–0.127	–0.083/–0.181
(33) I get nervous when the English teacher asks questions which I haven’t prepared in advance.	**0.560/0.264**	0.249/0.668	0.076/–0.020	0.313/0.167	0.300/0.197	–0.067/0.036

*Loadings of items included in corresponding factors are in bold.*

**TABLE A3:** Loadings of principal components of FLLAS (phases 1/2) [KMO = 0.915 in phase 1 and 0.895 in phase 2 (*p* ≤ 0.001)].

	Factor 1	Factor 2	Factor 3	Factor 4
(37) I get upset when I’m not sure whether I understand what I’m hearing in English.	**0.720/0.630**	–0.118/0.297	0.147/0.254	–0.076/0.082
(38) When I listen to English, I often understand the words but still can’t quite understand what the speaker is saying.	**0.435/0.309**	–0.124/0.190	0.416/0.602	0.299/0.098
(39) When I’m listening to English, I get so confused I can’t remember what I’ve heard.	0.583/0.678	–0.197/0.231	**0.367/0.289**	0.178/0.081
(40) I feel intimidated whenever I have a listening passage in English to listen to.	**0.688/0.790**	–0.268/0.174	0.098/0.119	0.113/–0.140
(41) I am nervous when I am listening to a passage in English when I’m not familiar with the topic.	**0.752/0.642**	–0.125/0.432	0.235/0.023	–0.004/0.192
(42) I get upset whenever I hear unknown grammar while listening to English.	**0.808/0.729**	–0.058/0.170	0.065/0.252	0.147/–0.066
(43) When listening to English I get nervous and confused when I don’t understand every word.	**0.761/0.750**	–0.129/0.146	0.048/0.101	0.049/0.053
(44) It bothers me to encounter words I can’t pronounce while listening to English.	**0.738/0.676**	–0.093/0.357	0.139/0.161	0.074/0.015
(45) I usually end up translating word by word when I’m listening to English.	0.464/0.433	–0.197/0.234	**0.352/0.487**	0.131/0.085
(46) By the time you get past the strange sounds in English, it’s hard to remember what you’re listening to.	0.431/0.493	–0.191/0.467	**0.554/0.330**	–0.095/0.296
(47) I am worried about all the new sounds you have to learn to understand spoken English.	**0.460/0.689**	–0.318/0.178	0.340/0.244	0.251/–0.201
(48) I enjoy listening to English.	–0.127/–0.261	**0.734/**–**0.567**	–0.118/–0.183	–0.222/0.495
(49) I feel confident when I am listening to English.	–0.229/–0.218	**0.774/**–**0.752**	–0.266/–0.184	–0.006/0.296
(50) Once you get used to it, listening to English is not so difficult.	–0.252/–0.067	**0.759/**–**0.329**	0.112/–0.204	–0.056/0.716
(51) The hardest part of learning English is learning to understand spoken English.	–0.021/0.505	–**0.038/0.013**	0.741/–0.117	0.243/0.358
(52) I would be happy just to learn to read English rather than having to learn to understand spoken English.	0.192/0.560	–**0.060/**–**0.355**	0.235/0.164	0.752/–0.010
(53) I don’t mind listening to English by myself but I feel very uncomfortable when I have to listen to English in a group.	**0.526/0.614**	–0.123/0.111	0.043/0.102	0.187/0.182
(54) I am satisfied with the level of listening comprehension in English that I have achieved so far.	–0.192/–0.216	**0.545/**–**0.787**	–0.416/–0.007	0.306/–0.016
(55) English culture and ideas seem very foreign to me.	0.389/0.075	–0.327/–0.056	–0.097/0.856	**0.407/**–**0.171**
(56) You have to know so much about English history and culture in order to understand spoken English.	0.272/0.190	0.380/0.098	0.403/0.201	–**0.264/0.704**

*Loadings of items included in corresponding factors are in bold.*

## References

[B1] Aida Y. (1994). Examination of Horwitz, Horwitz and Cope’s construct of foreign language anxiety: the case of students of Japanese. *Mod. Lang. J.* 78 155–168. 10.2307/329005

[B2] Aparicio M. Bacao F. Oliveira T. (2017). Grit in the path to e-learning success. *Comput. Hum. Behav.* 66 388–399. 10.1016/j.chb.2016.10.009

[B3] Arnold J. (2000). Seeing through listening comprehension exam anxiety. *TESOL Q.* 34 777–786. 10.2307/3587791

[B4] Bailey K. M. (1983). “Competitiveness and anxiety in adult second language learning: looking at and through the dairy studies,” in *Classroom Oriented Research in Second Language Acquisition*, eds Seliger H. W. Long M. H. (Rowley, MA: Newbury House Publishers, Inc), 67–103. 10.14746/ssllt.2020.10.1.4

[B5] Bekleyen N. (2009). Helping teachers become better English students: causes, effects, and coping strategies for foreign language listening anxiety. *System* 37 664–675. 10.1016/j.system.2009.09.010

[B6] Botes E. Dewaele J. M. Greiff S. (2020). The foreign language classroom anxiety scale and academic achievement: an overview of the prevailing literature and a meta-analysis. *J. Psychol. Lang. Learn.* 2 26–56. 10.52598/jpll/2/1/3

[B7] Boudreau C. MacIntyre P. Dewaele J. M. (2018). Enjoyment and anxiety in second language communication: an idiodynamic approach. *Stud. Second Lang. Learn. Teach.* 8 149–170. 10.14746/ssllt.2018.8.1.7

[B8] Bryson J. R. Andres L. (2020). Covid-19 and rapid adoption and improvisation of online teaching: curating resources for extensive versus intensive online learning experiences. *J. Geo. High. Educ.* 44 608–623. 10.1080/03098265.2020.1807478

[B9] Chastain K. (1975). Affective and ability factors in second-language acquisition. *Lang. Learn.* 25 153–161. 10.1111/j.14671770.1975.tb00115.x

[B10] Chen R. T.-H. (2010). *Knowledge and Knowers in Online Learning: Investigating the Effects of Online Flexible Learning on Student Sojourners.* Unpublished doctoral dissertation. Wollongong, NSW: University of Wollongong.

[B11] Cheng Y. Horwitz E. K. Schallert D. L. (1999). Language anxiety: differentiating writing and speaking components. *Lang. Learn.* 49 417–446. 10.1111/0023-8333.00095

[B12] Cheng Y.-S. (2004). A measure of second language writing anxiety: scale development and preliminary validation. *J. Second Lang. Writ.* 13 313–335. 10.1016/j.jslw.2004.07.001

[B13] Cohen J. (1988). *Statistical Power Analysis for the Behavioral Sciences*, 2nd Edn. New Jersey, NJ: Lawrence Erlbaum Associates.

[B14] Coman C. îru L. G. Mesean-Schmitz L. Stanciu C. Bularca M. C. (2020). Online teaching and learning in higher education during the Coronavirus Pandemic: students’ perspective. *Sustainability* 12:10367. 10.3390/su122410367

[B15] Coryell J. E. Clark M. C. (2009). One right way, intercultural participation, and language learning anxiety: a qualitative analysis of adult online heritage and nonheritage language learners. *Foreign Lang. Ann.* 42 483–504. 10.1111/j.1944-9720.2009.01037.x

[B16] Côté S. Gaffney C. (2018). The effect of synchronous computer-mediated communication on beginner L2 learners’ foreign language anxiety and participation. *Lang. Learn. J.* 49 1–12. 10.1080/09571736.2018.1484935

[B17] De Paepe L. Zhu C. Depryck K. (2018). Online Dutch L2 learning in adult education: educators’ and providers’ viewpoints on needs, advantages and disadvantages. *Open Learn.* 33 18–33. 10.1080/02680513.2017.1414586

[B18] Elkhafaifi H. (2005). Listening comprehension and anxiety in the Arabic language classroom. *Mod. Lang. J.* 89 206–220. 10.1111/j.1540-4781.2005.00275.x

[B19] Golchi M. M. (2012). Listening anxiety and its relationship with listening strategy use and listening comprehension among Iranian IELTS learners. *Int. J. Engl. Linguist.* 2 115–128. 10.5539/ijel.v2n4p115

[B20] Gong Y. Gao X. Li M. Lai C. (2020a). Cultural adaptation challenges and strategies during study abroad: new Zealand students in China. *Lang. Cult. Curriculum.* 10.1080/07908318.2020.1856129 [Epub ahead of print].

[B21] Gong Y. Guo Q. Li M. Lai C. Wang C. (2021). Developing literacy or focusing on interaction: New Zealand students’ strategic efforts related to Chinese language learning during study abroad in China. *System* 98:102462. 10.1016/j.system.2021.102462

[B22] Gong Y. Ma M. Hsiang T. P. Wang C. (2020b). Sustaining international students’ learning of Chinese in China: shifting motivations among New Zealand students during study abroad. *Sustainability* 12 6289–6302. 10.3390/su12156289

[B23] Gregersen T. (2020). Dynamic properties of language anxiety. *Stud. Second Lang. Learn. Teach.* 10 67–87.

[B24] Gregersen T. S. Horwitz E. K. (2002). Language learning and perfectionism: anxious and nonanxious language learners’ reactions to their own oral performance. *Mod Lang. J.* 86 562–570. 10.1111/1540-4781.00161

[B25] Gregersen T. Macintyre P. D. Meza M. D. (2014). The motion of emotion: idiodynamic case studies of learners’ foreign language anxiety. *Mod. Lang. J.* 98 574–588. 10.1111/modl.12084

[B26] Horwitz E. K. Horwitz M. B. Cope J. (1986). Foreign language classroom anxiety. *Mod. Lang. J.* 70 125–132. 10.2307/327317

[B27] Horwitz E. K. Tallon M. Luo H. (2010). “Foreign language anxiety,” in *Anxiety in Schools: The Causes, Consequences, and Solutions for Academic Anxieties*, ed. Cassady J. C. (New York, NY: Peter Lang), 95–115.

[B28] Hurd S. (2007). Anxiety and non-anxiety in a distance language learning environment: the distance factor as a modifying influence. *System* 35 487–508. 10.1016/j.system.2007.05.001

[B29] Hurd S. Xiao J. (2010). Anxiety and affective control among distance language learners in China and the UK. *RELC J.* 41 183–200. 10.1177/0033688210373640

[B30] Janse van Rensburg E. S. (2018). Effective online teaching and learning practices for undergraduate health sciences students: an integrative review. *Int. J. Afr. Nurs. Sci.* 9 73–80. 10.1016/j.ijans.2018.08.004

[B31] Jee M. J. (2016). Exploring Korean heritage language learners’ anxiety: ‘We are not afraid of Korean!’. *J. Multiling. Multicult. Dev.* 37 56–74. 10.1080/01434632.2015.1029933

[B32] Kaisar M. T. Chowdhury S. Y. (2020). Foreign language virtual class room: anxiety creator or healer? *Engl. Lang. Teach.* 13 130–139. 10.5539/elt.v13n11p130

[B33] Kim J. (2002). Anxiety and foreign language listening. *Engl. Teach.* 57 3–34.

[B34] Kleinmann H. H. (1977). Avoidance behavior in adult second language acquisition. *Lang. Learn.* 27 93–107. 10.1111/j.14671770.1977.tb00294.x

[B35] Kruk M. (2018). Changes in foreign language anxiety: a classroom perspective. *Int. J. Appl. Linguist.* 28 31–57. 10.1111/ijal.12182

[B36] Lei S. A. Gupta R. K. (2010). College distance education courses: evaluating benefits and costs from institutional, faculty and students’ perspectives. *Education* 130 616–631.

[B37] Liu H. J. Cheng S. H. (2014). Assessing language anxiety in EFL students with varying degrees of motivation. *Electronic J. For. Lang. Teach.* 11 285–299.

[B38] Liu M. (2006). Anxiety in Chinese EFL students at different proficiency levels. *System* 34 301–316. 10.1016/j.system.2006.04.004

[B39] Liu M. (2016). Interrelations between foreign language listening anxiety and strategy use and their predicting effects on test performance of high-and low-proficient Chinese university EFL learners. *Asia Pac. Educ. Res.* 25 647–655. 10.1007/s40299-016-0294-1

[B40] Liu M. (2018). Bilingual/multilingual learners’ willingness to communicate in and anxiety on speaking Chinese and their associations with self-rated proficiency in Chinese. *Int. J. Biling. Educ. Biling.* 21 54–69. 10.1080/13670050.2015.1127889

[B41] Liu M. Huang W. (2011). An exploration of foreign language anxiety and English learning motivation. *Educ. Res. Int.* 2011:493167. 10.1155/2011/493167

[B42] Liu M. Jackson J. (2008). An exploration of Chinese EFL learners’ unwillingness to communicate and foreign language anxiety. *Mod. Lang. J.* 92 71–76. 10.1111/j.1540-4781.2008.00687.x

[B43] Liu M. Xiangming L. (2019). Changes in and effects of anxiety on English test performance in Chinese postgraduate EFL classrooms. *Educ. Res. Int*. 2019:7213925. 10.1155/2019/7213925

[B44] Lu Z. Liu M. (2015). An investigation of Chinese university EFL learner’s foreign language reading anxiety, reading strategy use and reading comprehension performance. *Stud. Second Lang. Learn. Teach.* 5 65–85. 10.14746/ssllt.2015.5.1.4

[B45] O’Doherty D. Dromey M. Lougheed J. Hannigan A. Last J. McGrath D. (2018). Barriers and solutions to online learning in medical education—an integrative review. *BMC Med. Educ.* 18, 130–141. 10.1186/s12909-018-1240-0 29880045 PMC5992716

[B46] MacIntyre P. D. (2017). “An overview of language anxiety research and trends in its development,” in *New Insights into Language Anxiety: Theory, Research and Educational Implications*, eds Gkonou C. Daubney M. Dewaele J.-M. (Bristol: Multilingual Matters), 11–30. 10.21832/9781783097722-003

[B47] MacIntyre P. D. Gardner R. C. (1994). The subtle effects of language anxiety on cognitive processing in the second language. *Lang. Learn.* 44 283–305. 10.1111/j.1467-1770.1994.tb01103.x

[B48] MacIntyre P. D. Gregersen T. Mercer S. (2020). Language teachers’ coping strategies during the Covid-19 conversion to online teaching: correlations with stress, wellbeing and negative emotions. *System* 94:102352. 10.1016/j.system.2020.102352

[B49] Matsuda S. Gobel P. (2004). Anxiety and predictors of performance in the foreign language classroom. *System* 32 21–36. 10.1016/j.system.2003.08.002

[B50] Mills N. Pajares F. Herron C. (2006). A reevaluation of the role of anxiety: self-efficacy, anxiety, and their relation to reading and listening proficiency. *For. Lang. Ann.* 39 276–295. 10.1111/j.1944-9720.2006.tb02266.x

[B51] Oxford R. L. (2008). *Language Learning Strategies: What Every Teacher Should Know.* Beijing: Beijing World Publishing Corporation.

[B52] Pae T. I. (2013). Skill-based L2 anxieties revisited: their intra-relations and the inter-relations with general foreign language anxiety. *Appl. Linguist.* 34 232–252. 10.1093/applin/ams041

[B53] Phillips E. M. (1992). The effects of language anxiety on students’ oral test performance and attitudes. *Mod. Lang. J.* 76 14–26. 10.1111/j.1540-4781.1992.tb02573.x

[B54] Piniel K. Csizér K. (2015). “Changes in motivation, anxiety and self-efficacy during the course of an academic writing seminar,” in *Motivational Dynamics in Language Learning*, eds Dörnyei Z. MacIntyre P. Henry A. (Bristol: Multilingual Matters), 164–189. 10.21832/9781783092574-015

[B55] Scarcella R. C. Oxford R. L. (1992). *The Tapestry of Language Learning: The Individual in the Communicative Classroom.* Boston, MA: Heinle & Heinle Publishers.

[B56] Scull J. Phillips M. Sharma U. Garnier K. (2020). Innovations in teacher education at the time of COVID19: an Australian perspective. *J. Educ. Teach.* 46 497–506. 10.1080/02607476.2020.1802701

[B57] Sellers V. D. (2000). Anxiety and reading comprehension in Spanish as a foreign language. *For. Lang. Ann.* 33 512–520. 10.1111/j.1944-9720.2000.tb01995.x

[B58] Serraj S. Noordin N. B. (2013). Relationship among Iranian EFL students’ foreign language anxiety, foreign language listening anxiety and their listening comprehension. *Engl. Lang. Teach.* 6 1–12. 10.5539/elt.v6n5p1

[B59] Shirvan M. E. Taherian T. (2021). Longitudinal examination of university students’ foreign language enjoyment and foreign language classroom anxiety in the course of general English: latent growth curve modeling. *Int. J. Biling. Educ. Biling.* 24 31–49. 10.1080/13670050.2018.1441804

[B60] Singh V. Thurman A. (2019). How many ways can we define online learning? A systematic literature review of definitions of online learning (1988-2018). *Am. J. Distance Educ.* 33 289–306. 10.1080/08923647.2019.1663082

[B61] Spielberger C. D. (1983). *Manual for the State-Trait Anxiety Inventory.* Palo Alto, CA: Consulting Psychologists Press.

[B62] Vandergrift L. (2004). Listening to learn or learning to listen? *Annu. Rev. Appl. Linguist.* 24 3–25. 10.1017/S0267190504000017

[B63] Wang K. Zhang L. Ye L. (2020). A nationwide survey of online teaching strategies in dental education in China. *J. Dent. Educ.* 85 1–7. 10.1002/jdd.12413 32954532 PMC7537095

[B64] Wang S. (2010). An experimental study of Chinese English major students’ listening anxiety of classroom learning activity at the university level. *J. Lang. Teach. Res.* 1 562–568. 10.4304/jltr.1.5.562-568

[B65] Wang S.-Y. Cha K.-W. (2019). Foreign language listening anxiety factors affecting listening performance of Chinese EFL learners. *J. Asia TEFL* 16 121–134. 10.18823/asiatefl.2019.16.1.8.121

[B66] Xiangming L. Liu M. Zhang C. (2020). Technological impact on language anxiety dynamic. *Comput. Educ.* 150:103839. 10.1016/j.compedu.2020.103839

[B67] Xu J. Huang Y. T. (2018). The mediating effect of listening metacognitive awareness between listening test anxiety and listening test performance. *Asia Pac. Educ. Res.* 27 313–324. 10.1007/s40299-018-0388-z

[B68] Young D. J. (1986). The relationship between anxiety and foreign language oral proficiency ratings. *For. Lang. Ann.* 19 439–445. 10.1111/j.1944-9720.1986.tb01032.x

[B69] Zhang X. (2013). Foreign language listening anxiety and listening performance: conceptualizations and causal relationships. *System* 41 164–177. 10.1016/j.system.2013.01.004

